# Consonance Shapes the Multisensory and Emotional Mappings of Musical Intervals Across English‐ and Mandarin‐Speakers

**DOI:** 10.1111/nyas.70337

**Published:** 2026-07-23

**Authors:** Nicola Di Stefano, Alessandro Ansani, Andrea Schiavio, Suvi Saarikallio, Petri Toiviainen, Elvira Brattico, Charles Spence

**Affiliations:** ^1^ Institute of Cognitive Sciences and Technologies National Research Council Rome Italy; ^2^ Centre of Excellence in Music, Mind, Body and Brain—Department of Music, Art and Culture Studies University of Jyväskylä Jyväskylä Finland; ^3^ School of Arts and Creative Technologies University of York York UK; ^4^ Center for Music in the Brain, Department of Clinical Medicine Aarhus University Aarhus Denmark; ^5^ The Royal Academy of Music, Aarhus/Aalborg Aarhus Denmark; ^6^ Department of Education, Psychology, Communication University of Bari Aldo Moro Bari Italy; ^7^ Department of Experimental Psychology University of Oxford Oxford UK

**Keywords:** consonance, crossmodal associations, dissonance, gradus suavitatis, multisensory

## Abstract

The exploration of sound's multisensory attributes has become increasingly central to the cognitive science of music, yet research has largely focused on a limited set of sensory dimensions, often within a single modality. Moreover, the extent to which these associations are consistently perceived by listeners from different countries and linguistic backgrounds remains an open question. To address this issue, we conducted a comprehensive investigation of how two groups of listeners—146 English‐speakers from North America and the United Kingdom and 64 Mandarin‐speakers from China—map harmonic intervals onto 23 sensory and affective dimensions spanning vision, touch, taste, and emotion. Our results revealed consistent associations across groups between musical intervals and gustatory, tactile, visual, and emotional attributes. Importantly, these multisensory mappings clustered according to the consonance/dissonance dimension: consonant intervals were rated as more sweet, smooth, warm, round, symmetric, and bright, whereas dissonant intervals were rated as more rough, cold, spiky, asymmetric, and dark. Crucially, country of origin and language had minimal influence on these mappings, as statistical analysis based on model comparisons overwhelmingly favored simpler models without group interaction terms. These findings suggest that consonance–dissonance may serve as a core organizing principle underlying multisensory mappings of musical intervals.

## Graphical abstract

We investigated how English‐speakers from North America and the United Kingdom and Mandarin‐speakers from China map musical intervals onto sensory and emotional dimensions. Across vision, touch, taste, and affect, consonant intervals were consistently linked to positive, smooth, warm, and bright qualities, whereas dissonant intervals were associated with negative, rough, cold, and dark ones. These robust crossmodal associations were observed in both groups of participants, highlighting consonance–dissonance as the shared organizing principle driving the multisensory mappings.

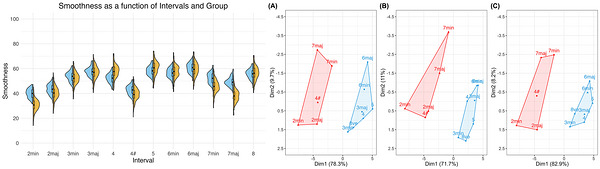

## 1 Introduction

The past few decades have seen a surge of interest in the multiple sensory dimensions of sound across cognitive science. Studies have extensively explored cross‐sensory associations between auditory percepts and non‐auditory features in typical (i.e., non‐synesthetic) individuals to better understand the underlying mechanisms of perception, the integration of sensory information, and the cognitive and affective processes that shape how we experience and interpret the world. The available evidence demonstrates that these perceptual phenomena—commonly referred to as “crossmodal associations” or “crossmodal correspondences” [1]—are consistently observed across individuals [2, 3].

Studies have primarily investigated how various musical features—such as pitch, timbre, mode, loudness, rhythm, and harmony—are associated with visuospatial properties like brightness, lightness, and elevation [4, 5, 6, 7, 8, 9, 10, 11]. For instance, increases in pitch are linked to brighter visual stimuli [7] and higher spatial elevation [4]. Notably, many of these correspondences have been observed across remote cultures and languages. For instance, the correspondence between pitch–height has been observed in a tribe from Cambodia [12] and the bouba/kiki effect in a population from Namibia [13].

Expanding beyond basic auditory features, Maimon et al. [6] found that tonal stability—a concept related to the degree to which pitches and chords are perceived as anchored, resolved, or central within a tonal framework—is also associated with visual brightness (see also the work by Maimon et al. [14], for links between tonal and spatial relationships). Additionally, more consonant intervals have been linked to cooler, softer, more transparent, and less arousing color patches [15].

Researchers have explored auditory‐based associations beyond the spatial senses, documenting a variety of mappings between auditory stimuli and gustatory/flavor stimuli [16, 17]. Studies consistently show that higher pitched sounds are associated with sweet and sour tastes, whereas lower pitched sounds are linked to bitter tastes [18, 19, 20]. Harmonic properties have also been examined, with consonant chords typically associated with sweet tastes and dissonant chords with sour tastes [21, 22].

Mesz et al. [23] conducted two experiments investigating taste–music associations. In the first one, musicians were invited to improvise using a MIDI keyboard with piano timbre on the basis of four taste words: sweet, sour, bitter, and salty. The analysis revealed consistent musical patterns—“bitter” improvisations were low‐pitched and legato, “salty” improvisations were staccato, “sour” improvisations were high‐pitched and dissonant, and “sweet” improvisations were consonant, slow, and soft. In a second experiment, non‐musicians listened to excerpts from these improvisations and, in a forced‐choice task, tried to identify which taste word each piece represented. The results revealed that participants identified the correct taste word at rates significantly above chance. Further research has explored auditory–olfactory crossmodal correspondences, with findings showing that higher pitched sounds (∼G4) are matched with scents like candied orange and iris flower, whereas lower pitched sounds (∼G3) are associated with odors such as musk and roasted coffee [24].

Although most studies have used verbal stimuli to evoke sensory features, Murari et al. [25] used physical materials such as wood and polystyrene foam to represent hardness and softness, sandpaper to convey smoothness and roughness, bitter and sweet liquids for gustatory contrasts, and cold and hot water for temperature variations. Additionally, they selected excerpts from the Western classical repertoire (e.g., Vivaldi, Bach, Mozart, and Wagner) as auditory stimuli. The results of their study revealed several consistent patterns of crossmodal associations among participants, with certain sensory qualities—such as smooth/rough and sweet/bitter—distinguishing musical excerpts more effectively than others, such as soft/hard (see Di Stefano and Spence [26] for a multisensory review on roughness).

Another strand of research has explored the association between musical intervals and emotional descriptors [5, 27, 28]. For example, Costa et al. [29] invited participants to rate harmonic intervals (i.e., combinations of two tones simultaneously played defined in terms of the number of scale steps separating the tones) using 30 adjectives commonly associated with musical expressiveness, such as cheerful/cheerless, serene/gloomy, and quiet/restless. Their findings revealed significant effects of both register and consonance/dissonance. Participants tended to rate low‐pitched and dissonant intervals more negatively, whereas higher pitched and consonant intervals were rated more positively.

Despite a growing body of research on crossmodal associations involving musical stimuli, most studies have focused on a narrow range of sensory features—typically within one or two domains—and have used diverse methodologies, limiting the comparability and generalizability of findings. Additionally, the extent to which these associations remain consistent across participants from different countries, or continents, and with different linguistic backgrounds is largely unexplored. To advance current understanding, the present study systematically examines how musical intervals are associated with a broad set of sensory and affective dimensions in a group of English‐speakers from North America and the United Kingdom and another group of Mandarin‐speakers from China. In doing so, we aim to contribute to ongoing debates in music cognition, multisensory perception, and the foundations of crossmodal correspondences.

## 2 Materials and Methods

### 2.1 Participants

We recruited 210 participants through Prolific.com (*M*_age_ = 37.40 years, *SD* = 13.66, 61.42% females, 2 did not disclose their gender). One group was composed of 146 English‐speakers mainly from the United Kingdom (87%), United States (5%), and Canada (5%) (*M*_age_ = 40.26 years, *SD* = 14.52, 57.30% females, one did not disclose their gender). Another group was composed of 64 Mandarin‐speakers from China (*M*_age_ = 30.89 years, *SD* = 8.50, 68.75% females, one did not disclose their gender).

Information about musical background was collected using both subjective and objective measures. With regard to self‐reported musical expertise, as assessed by the single‐item measure proposed by Zhang and Schubert [30], 47.6% defined themselves as music‐loving non‐musicians, 29.0% non‐musicians, 15.7% amateur musicians, 4.3% serious amateur musicians, 1.9% semi‐professional musicians, and 1.4% professional musicians. The distribution of expertise categories did not differ significantly between the two samples, as indicated by a chi‐square test, *χ* ^2^(5, *N* = 210) = 7.04, *p* = 0.218.

To obtain a more quantitative assessment of musical expertise, we administered two subscales of the Goldsmith Musical Sophistication Index [31], namely, Musical Training (i.e., amount of formal musical training received) and Emotional Engagement with Music (i.e., ability to talk about emotions that music expresses). Both subscales had good reliability in our samples (*α*_MT‐West_ = 0.92, 95% CI [0.90, 0.94]; *α*_Emo‐West_ = 0.74, 95% CI [0.66, 0.80]; *α*_MT‐East_ = 0.84, 95% CI [0.78, 0.88]; *α*_Emo‐West_ = 0.79, 95% CI [0.64, 0.87]). The samples were not statistically different in terms of emotional engagement with music (*U* = 4243, *p* = 0.289, *r*_rb_ = 0.09, 95% CI [−0.07, 0.25]), but the Eastern sample showed higher musical training (*U* = 6209, *p* < 0.001, *r*_rb_ = 0.33, 95% CI [0.17, 0.47]).

### 2.2 Musical Stimuli

Using the piano Noire pure VST instrument (see https://www.native‐instruments.com/fileadmin/ni_media/downloads/manuals/NOIRE_Manual.pdf for further information about the sampling), 144 auditory stimuli (wav, 24‐bit, 44.1 kHz) were generated consisting of two piano notes played simultaneously, that is, harmonic intervals. Starting from the G below middle C (G_3_, 195.9 Hz) up to F#/G♭_4_ (369.9 Hz), we recorded all of the dyads from the minor second to the octave. In this process, each note in the range has been used as the root note, and all the above‐mentioned intervals were generated from each root note. Therefore, the full pitch range was G_3_–F#/G♭_5_. The stimuli were identical in terms of dynamics (velocity MIDI value = 100, corresponding to a *forte*) and duration (2 s).

### 2.3 Sensory and Emotional Features

We selected 23 sensory and emotional/affective features. The sensory features spanned three domains: visuospatial, tactile, and gustatory. Visuospatial features included far/close, left/right, low/high, small/big, dark/bright, *maluma/takete* (i.e., curved and angular shapes, respectively), colorful/black and white, warm/cold hue, and symmetric/asymmetric. Tactile features included heavy/light, soft/hard, rough/smooth, and cold/hot (note that roughness has been conceived of as a sensory feature that can be associated with visual, auditory, and, to a lesser extent, gustatory stimuli [26]). Gustatory features included spicy, sweet, salty, bitter, and umami. Additionally, we incorporated five pairs of opposite emotional/affective adjectives: sad/happy, pleasant/unpleasant, tender/tough, dominant/submissive, and arousing/relaxing.

### 2.4 Procedure

In an online procedure administered via Qualtrics, participants were presented with all 12 harmonic intervals, randomly selected from the sample of 144 stimuli. Each harmonic interval was presented once to each participant, and randomization ensured that all of the intervals were presented in a random order. Western participants completed the procedure in English, whereas Eastern participants completed it in Mandarin Chinese.

Participants were invited to rate each audio stimulus for all of the 23 sensory and affective dimensions using a 100‐point visual analog scale (VAS). For bipolar features, the scale presented the opposite adjectives at the extreme of the scale (e.g., far/near). For unipolar features (e.g., bitter), only one term was presented close to one end of the VAS. During the rating task, participants were allowed to replay each interval as many times as needed. The median duration of the procedure was 22.49 min (*SD* = 12.08). The protocol was approved by the Research Ethics and Integrity Committee of the National Research Council of Italy (no. 0323801).

### 2.5 Statistical Analysis

All analyses were performed on RStudio (2024.04.2). To model the differences of the intervals across all dimensions, a generalized linear mixed model (GLMM) approach was carried out via the *lme4* package [32]. The participants’ and the stimuli's keys were modeled as random intercepts to account for their variability [33]. The following formula was used:

Dimensionvalue=interval+(1|ID)+(1|Key)

In greater detail, as the distribution of several of our dependent variables diverted from normality, two instances of every model were built: a general (i.e., Gaussian) version and a generalized version, wherein a gamma distribution was used with a logarithmic link function [34]. The two instances were compared in terms of Akaike and Bayesian information criteria (AIC and BIC) and Bayes factors (BFs) computed via BIC approximation (see next paragraph). Except for the gustatory dimensions, which showed a better fit with the gamma‐distributed generalized models, all the other dimensions showed a significantly better fit with the Gaussian model. The reported significance levels derive from the instance with the best fit.

To inspect the role of the group (i.e., Western and Eastern), a model comparison approach was used [35]. Each model (one per dimension) was fit into two versions: simple and complex. In the simple model, the interval was the only predictor; in the complex model, an interaction term *interval × group* was added. Subsequently, the models were compared based on BIC and AIC. Moreover, BFs were computed with BIC approximation. (Given the high number of observations (*N* = 2520), likelihood ratio tests (LRTs) were avoided because they do not penalize model complexity. Furthermore, LRT is inherently biased toward more complex models, as the likelihood always increases with additional parameters. Moreover, LRT does not account for sample size, making it unsuitable for evaluating model parsimony). Such BFs represent a ratio of the relative evidence for the simpler model over the complex and answer the question “Under which model are the observed data more probable?”. They are computed as follows: $BF_{10}=e^{(\mathrm{BI}C_{0}-\mathrm{BI}C_{1})/2}$ where 1 and 0 represent the complex and simple models, respectively [36].

This procedure was conducted to assess whether the addition of the interaction term improved the fit significantly, thus confirming the importance of the group. Notably, the two metrics behave differently when it comes to penalization. Although AIC penalization is 2k (k being the number of parameters being estimated), BIC penalization is larger because it explicitly takes into account the number of observations, namely, ln(n)k. The former is therefore more liberal and suitable for prediction purposes, whereas the other's protection against overfitting makes it more conservative and optimal for inference purposes [37].

Subsequently, through the *emmeans* package [38], we computed the estimated marginal means (EMMs) of all intervals and performed deviation contrasts (also known as sum contrasts [39]) for the best‐performing model configuration. Thus, we compared the EMM of each interval against the grand mean of the dimension at hand. To control for multiple comparisons, the contrasts were adjusted through Bonferroni correction (i.e., for 12 tests).

Lastly, a *K*‐means algorithm was used to generate five alternative cluster solutions (range: 2–6). Several indices were computed with *NbClust* [40] to select the correct number of clusters: gap statistic [41], silhouette [42], KL7 [43], CH [44], and SD validity index [45]. Multidimensional scaling (MDS) was performed using the *factoextra* package [46].

As an attempt to quantify the degree of invariance between the two groups, we computed the cosine similarity between the two matrices of mean ratings (12 intervals × 23 dimensions). Cosine similarity assesses the alignment (angular similarity) of multivariate profiles rather than absolute differences in magnitude, making it appropriate for evaluating pattern‐level invariance across groups [47]. As not all variables were measured on the same scale (e.g., hue ranges from 0 to 2800 pixels), all dimensions were *z*‐transformed prior to the computation, thus ensuring that each variable contributed equally to the similarity metric. The cosine similarity was then computed on the vectorized standardized matrices.

In addition, to explore the geometric organization of the sensory and affective dimensions in a two‐dimensional space, a principal component analysis (PCA) was conducted. Prior to the PCA, all the ratings were *z*‐transformed within participants, thus ensuring an equal contribution to the analysis by all the participants. The PCA was conducted separately for the English‐ and Mandarin‐speakers’ groups, as well as for the full sample. Our focus was not on component retention or variance explanation, but rather on visualizing the relative positioning of the dimensions in the space defined by the first two principal components. For visualization, we plotted the first two components and their squared cosine values (cos^2^), which indicate the quality of representation of each score on the selected components.

## 3 Results

### 3.1 Model Comparison

According to the BIC, the simpler model is always preferable. In other words, the fit improvement of the models, which consider the group (i.e., Western/Eastern), is not sufficient to justify the group's role in predicting participants’ ratings. When considering the BFs, the evidence for the simpler models is overwhelming, the smallest BF being 6957, and the largest being 3.60 × 10^18^ (see Table S1).

When considering the AIC metric, however, in 9 out of 23 cases the complex model is preferred. This means that, in such models, the ratings of some of the intervals are different in the two samples. It is worth stressing that a statistically significant interaction does not necessarily imply substantive or theoretical relevance, particularly in the context of model comparison. Although the inclusion of the group interaction term leads to a better fit according to the AIC, this does not inherently mean that the interaction effect is noteworthy. The discrepancy between AIC and BIC in model selection underscores the importance of considering model complexity alongside interpretability. Given that BIC applies a stricter penalty for additional parameters, its lack of support for the more complex model suggests that the interaction effect may be an artifact of model flexibility rather than a meaningful effect. Thus, statistical significance alone should not be conflated with practical or theoretical importance, particularly in the presence of multiple comparisons and high‐dimensional estimation. Although many of the observed differences appear minor and not readily interpretable, the variation in ratings of “perfect” consonances (e.g., see Figures S1 and S4) may plausibly relate to their distinct functional roles in Western tonal and traditional Chinese music. Consistent with the model comparison results, we will describe the estimates stemming from the simple version of all models. However, according to the guidelines provided by Burnham and Anderson [48, p. 71], it was deemed necessary to report in the Supporting Information the graphs of the features whose evidence for the complex model was defined as substantial (i.e., ΔAIC > 10, see Figures S1–S7).

### 3.2 Modeling Results

**FIGURE 1:**
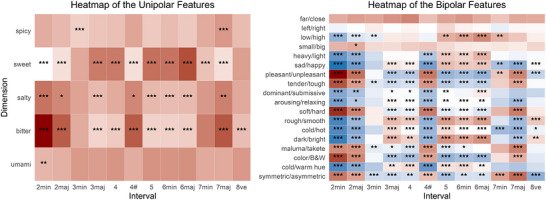
Left. Heatmap of the unipolar features. Hues vary according to estimated marginal mean (EMM) values, with higher EMM values being represented by more saturated colors. Right. Heatmap of the bipolar features. Hues vary according to EMM values, with higher EMM values being represented by more saturated colors. Blue/red colors represent the feature at the left/right side of the bipolar continuum (e.g., far is blue and close is red). Significant relationships between intervals and unipolar/bipolar features are marked with “*,” that is, the EMM of the interval is significantly higher/lower than the grand mean of the same dimension. To provide details on the significance, we added *** for *p* < 0.001, ** for 0.001 < *p* < 0.009, and * for 0.010 < *p* < 0.049. Empty cells indicate the absence of the deviation contrast significance.

The results of the GLMMs indicate significant relationships between many sensory and affective features and musical intervals (see Figure 1). In the following sections, we explore these dimensions in greater detail, focusing on gustatory, spatial and physical, emotional and affective, tactile, and color and shape associations.

#### 3.2.1 Gustatory

The intervals did not differ in the umami dimension, except for the minor second, which was rated significantly less umami than the average. In terms of spiciness, minor third intervals were rated as not spicy, whereas the major seventh is associated with higher values of spiciness. The pattern of the salty dimension presents high values for seconds, tritone, and major sevenths and low values for major thirds, perfect fifths, and sixths. The minor second is perceived as the most bitter interval, followed by major seventh, tritone, and major seconds. Major third, fifth, and minor sixth were the least bitter intervals. The dimension of sweetness showed an opposite pattern, with major sixth being the sweetest interval, followed by fifth, minor sixth, major third, and perfect fourth. The least sweet were minor second, tritone, major second, and major seventh.

#### 3.2.2 Spatial and Physical Attributes

No differences were found in the far/close and left/right dimensions. Low/high ratings seem to be affected by the size of the interval, with the seconds and minor third intervals being associated with the low end of the spectrum, whereas the fifth, sixths, and major seventh are associated with the high end of the spectrum. The same intervals were also rated as the lightest ones (except for the major seventh). The minor seconds were also rated as bigger and heavier. Concerning size, the major second appears to be the only “big” interval. Finally, the tritone was perceived as heavy.

#### 3.2.3 Emotional/Affective

All emotional and affective features polarize intervals. Negatively valenced features (e.g., sad, unpleasant, tough, arousing, dominant) tend to be associated with seconds, tritones, and sevenths, while positively valenced ones (e.g., happy, pleasant, tender, relaxing, submissive) with major thirds, perfect fourths and fifths, sixths, and octaves.

#### 3.2.4 Tactile

**FIGURE 2:**
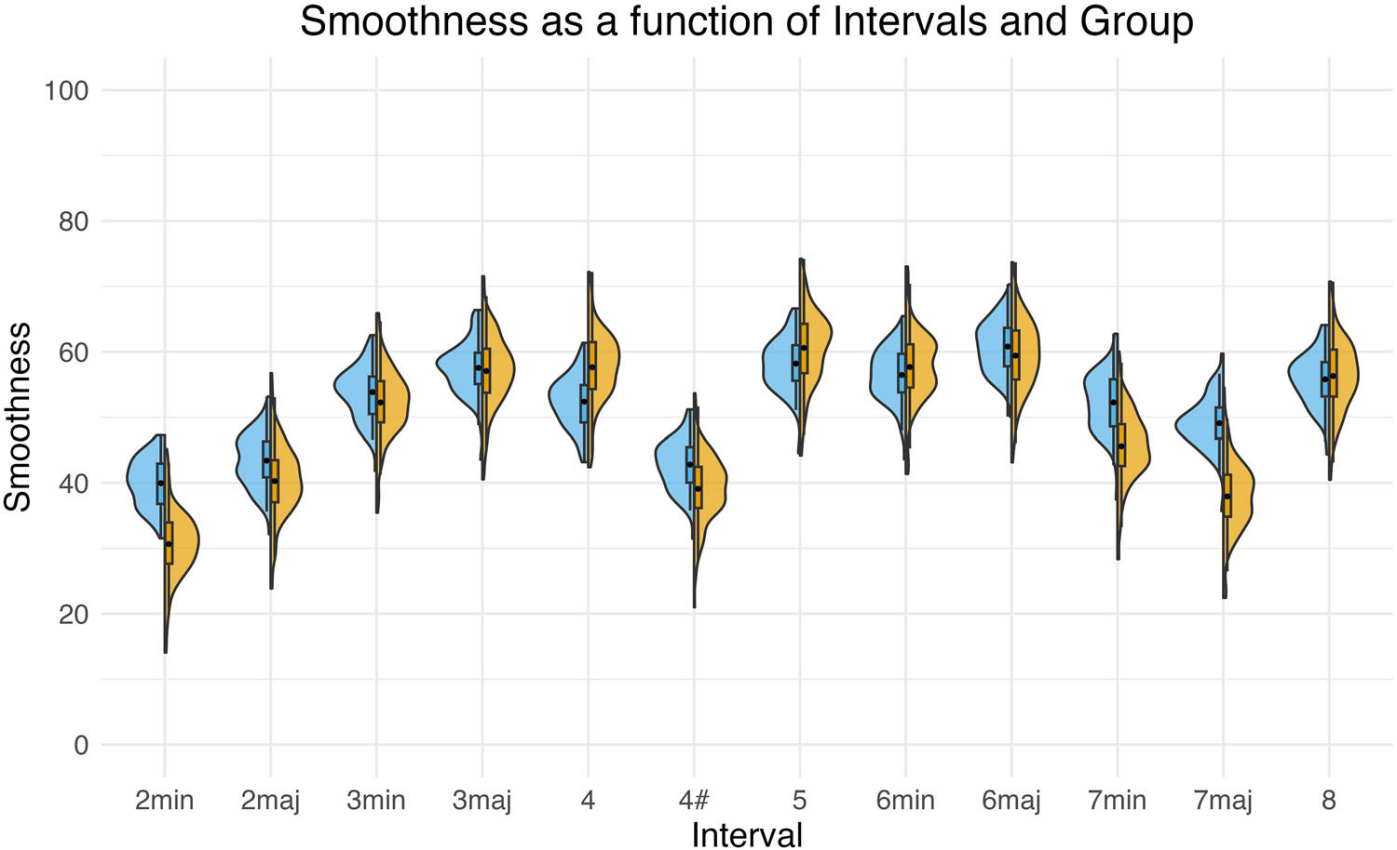
Smoothness as a function of intervals and group. On the *y*‐axis, a value of 100 indicates that participants rated the interval as smooth, whereas 0 indicates that the interval is perceived as rough. The form of the violin plots indicates the distribution curve. The boxplots within each violin represent interquartile ranges (IQRs). Black dots within the boxplots indicate mean values. Colors represent groups, namely, Mandarin‐speakers (blue) and English‐speakers (orange).

The dimensions in the tactile domain showed great consistency in terms of the association patterns across the eight intervals. Minor second, tritone, and major seventh were judged as harder, rougher, and colder. Conversely, major third, fourth, fifth, and major sixth were deemed softer, smoother (see Figure 2), and hotter. Octaves and minor sixths were also judged as smooth (see Figure 2), the latter being also perceived as soft. Finally, minor sevenths were perceived as cold.

#### 3.2.5 Color and Shape

**FIGURE 3:**
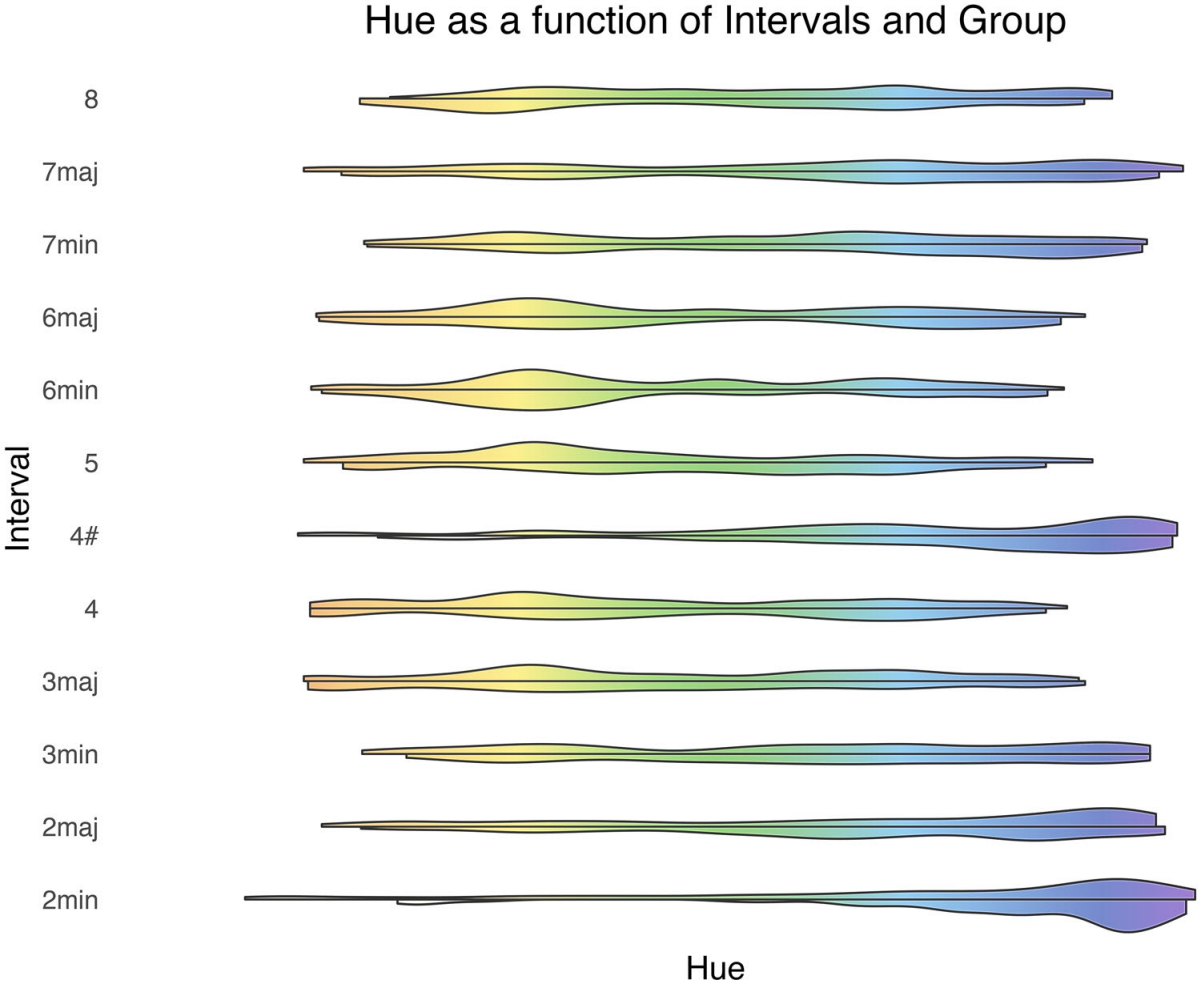
Hue as a function of intervals and group. Split violin plots represent the hue distribution across intervals. The density estimation was performed using kernel density estimation (KDE) with a bandwidth adjustment factor of 0.5 to provide a more localized representation of the data distribution. The violin plots were trimmed and scaled to ensure equal area across conditions. Different from the other figures, in this one, the observed (not predicted) values are plotted. However, to reduce the influence of extreme values, the dataset was filtered by removing observations below the 10th percentile and above the 90th percentile within each interval separately, preserving the relative distribution within each group. The upper split violin represents the English‐speakers’ sample; the lower one pertains to the Mandarin‐speakers’ sample.

A high degree of consistency across participants was found in the color (see Figure 3) and shape domain. The seconds and tritone were consistently perceived as dark, spiky (*takete*), B&W, asymmetrical, and with a cold hue. The major seventh had the same associations except for the cold hue. In contrast, major thirds, fourths, fifths, and sixths were judged as bright, round (*maluma*), colorful, symmetrical, and with a warm hue. The major sixth lacked significance in the roundness dimension, whereas the minor third was perceived as round and symmetric. The minor seventh was asymmetric, whereas the octave was symmetric. Interestingly, dissonances tend to be associated more likely with blue hues (e.g., sevenths, tritone, and seconds), whereas consonances with yellowish ones (octaves, sixths, perfect fourths and fifths, and thirds).

### 3.3 Cluster Analysis, MDS, and Cosine Similarity

**FIGURE 4:**
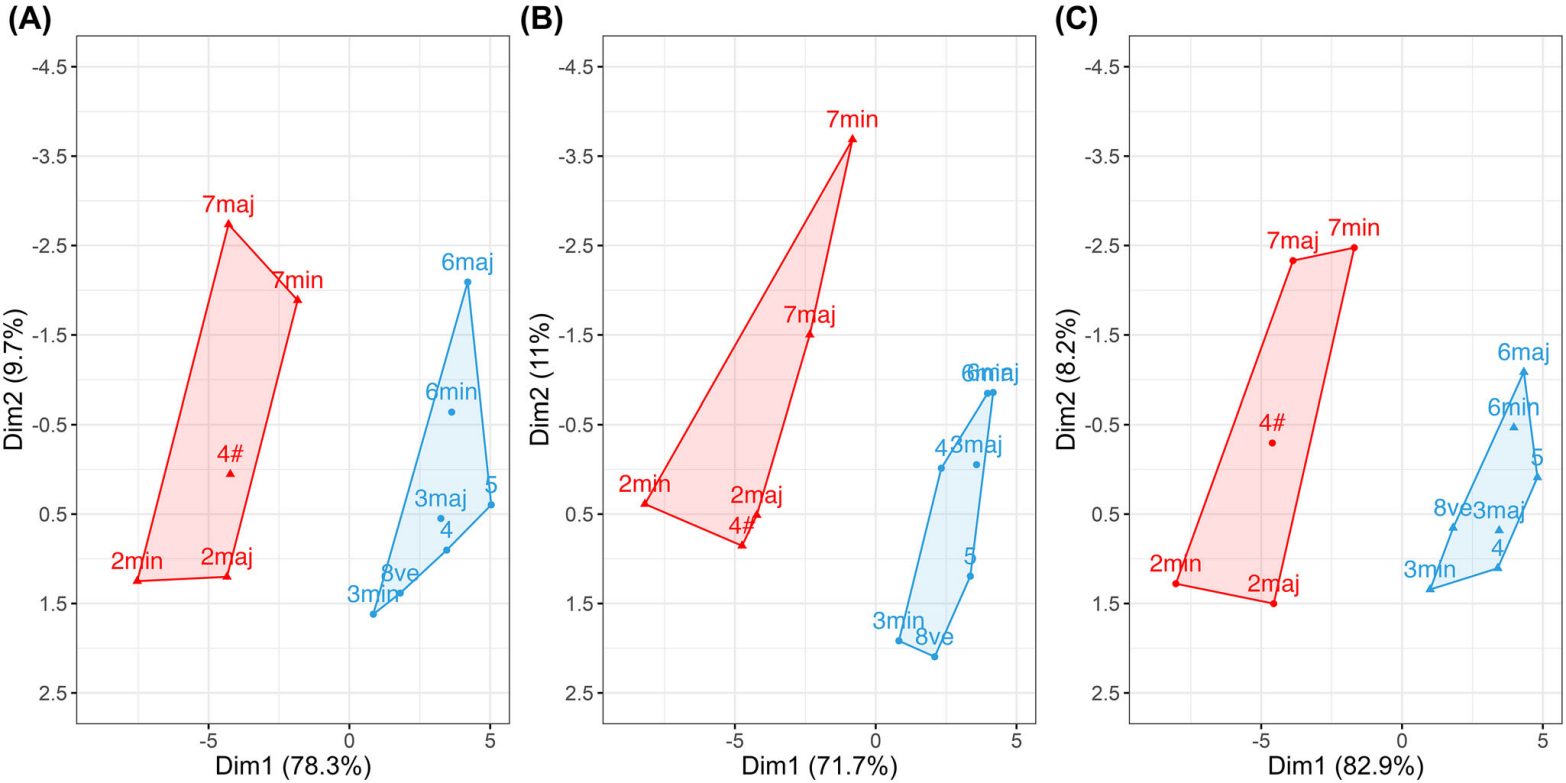
Multidimensional scaling (MDS) solutions for English‐speakers (A), Mandarin‐speakers (B), and overall samples (C). In all solutions, all the dissonant intervals appear to be in Cluster 1 (red), whereas the consonant intervals group to Cluster 2 (blue). Note that in the Mandarin‐speakers sample, the major and minor sixth intervals overlap.

We noticed that the patterns of participants’ ratings seemed to differentiate between consonant and dissonant intervals. To further inspect such a claim, we ran a cluster analysis. As an additional confirmation of the non‐determinant role of the group, three cluster analyses were performed on the English‐speakers, Mandarin‐speakers, and complete samples, including all of the 23 sensory/emotional features. In all cases, all of the indices converged on a clear two‐cluster solution. In Figure 4, we present the MDS representation for all samples.

For the sample who ran the experiment in English, seven intervals fell into Cluster 1 (within cluster sum of squares—WCSS = 38.10, in blue in Figure 4): minor third, major third, perfect fourth, perfect fifth, minor sixth, major sixth, and octave. Cluster 2 (WCSS = 43.67, in red in Figure 4) was composed of the remaining intervals: minor second, major second, tritone, minor seventh, and major seventh. The ratio of the between‐cluster sum of squares to the total sum of squares was 67.70%, thus indicating a strong separation between the clusters.

In the Mandarin‐speakers sample, the solution was overlapping, in that the same intervals fell into Cluster 1 (WCSS = 47.44) and Cluster 2 (WCSS = 59.23). The ratio of the between‐cluster sum of squares to the total sum of squares was 57.81%. When considering the whole sample, Cluster 1 (WCSS = 28.90, in blue in Figure 4) and Cluster 2 (WCSS = 44.42) appear well differentiated. The ratio of the between‐cluster sum of squares to the total sum of squares was 71.03%.

The cosine similarity computed between the two matrices of mean ratings (12 intervals × 23 dimensions) was 0.71. Although no universal thresholds exist, values higher than 0.70 are commonly interpreted as moderate‐to‐high degree of structural similarity in high‐dimensional settings, thus supporting the similarity between the patterns of the two samples. It is worth noting that, due to the *z*‐transformation of the scores, cosine similarity is mathematically equivalent to Pearson's correlation (*r* (274) = 0.71, 95% CI [0.65, 0.77], *p* < 0.001, see Figure S8).

Interestingly, in all samples, the cluster differentiation appears to overlap with the differentiation between consonant and dissonant intervals. Such a result is of particular interest if we consider that nowhere in the experimental procedure was the concept of consonance/dissonance mentioned, nor was it implied. Furthermore, when inspecting the *x* and *y* coordinates of the MDS solutions, it is quite straightforward to interpret their intrinsic sense. The intervals seem to be ordered on the *x*‐axis from the most dissonant to the most consonant. For this reason, we correlated the *x* Cartesian coordinates of the intervals with two indices of consonance/dissonance, namely, roughness and *gradus suavitatis*.

Roughness was calculated using two different metrics, one audio‐based and the other symbolic. For the audio‐based, we used Sethares’ model [49] as implemented in the MIR toolbox [50]. The process begins with extracting spectral peaks from the audio signal to identify prominent frequency components. Roughness is then computed for each frequency pair using the Plomp–Levelt curve [51], which models dissonance based on frequency separation. The total roughness is obtained by summing these values, weighted by the amplitudes of the peaks.

For the symbolic metric, we used the Hutchinson and Knopoff [52] model as implemented in the *incon* R package [53]. This model computes sensory dissonance from symbolic pitch representations by estimating interference between partials of the component tones according to critical‐band interactions derived from the Plomp–Levelt framework. Unlike the Sethares implementation, which operates directly on the acoustic signal and therefore incorporates timbral and spectral properties of the stimuli, the Hutchinson and Knopoff model relies on abstract pitch relationships and harmonic structures.

*Gradus suavitatis* (degree of agreeableness), proposed by Leonhard Euler [54], quantifies the consonance of an interval based on the simplicity of its frequency ratio, assuming just intonation [55]. For an interval ratio *p/q*, it is defined as 1 + Σ*_i_e_i_* (*r_i_ *− 1), where *r_i_* are the prime factors with multiplicity *e_i_* of the least common multiple of *p* and *q*. Lower values indicate greater consonance, as simpler frequency ratios are associated with more agreeable auditory perception.

**FIGURE 5:**
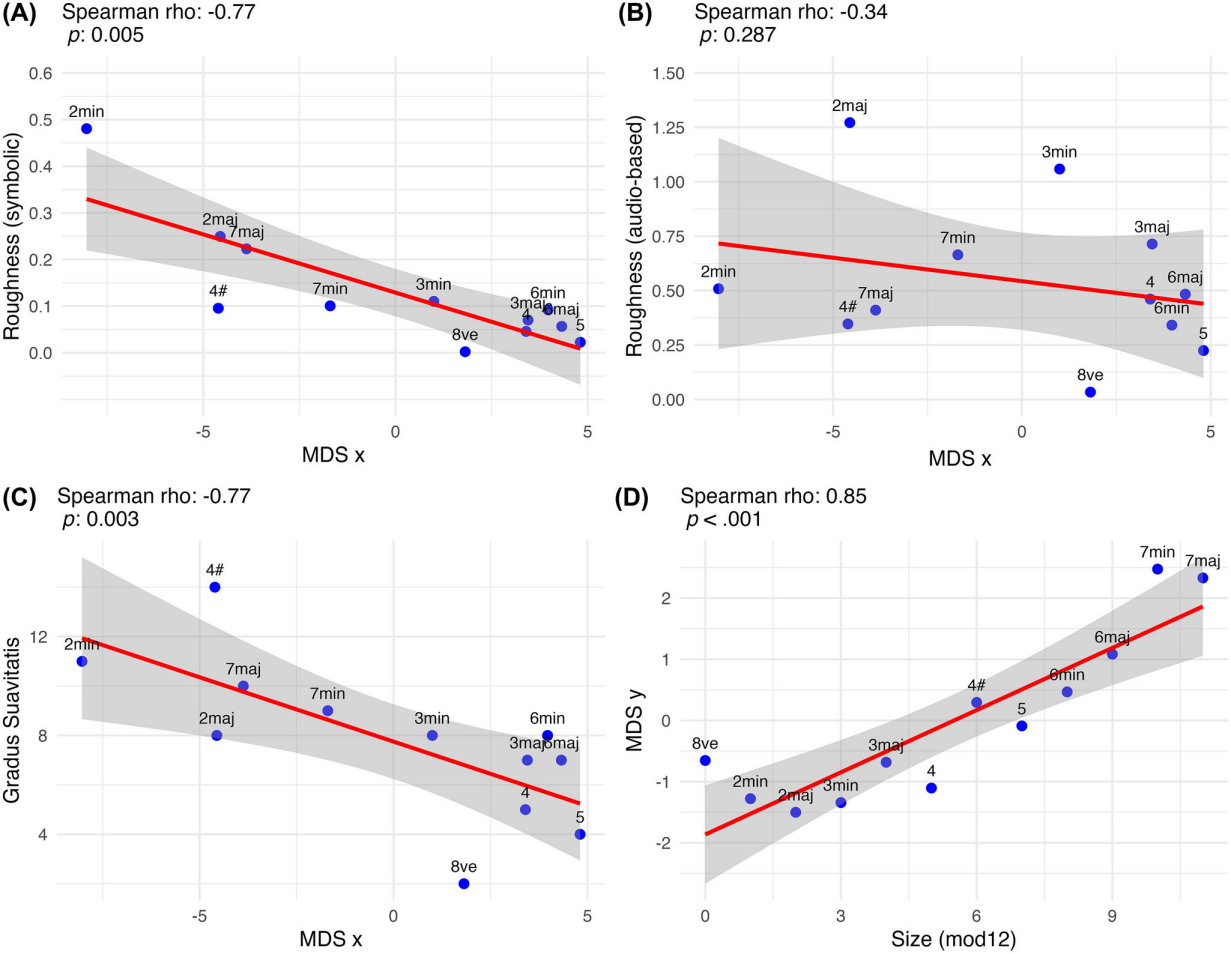
Correlation between the *x*‐axis of multidimensional scaling (MDS) and roughness as computed by the Hutchinson and Knopoff (1978) model (A) and by the Sethares [104] model (B). Correlation between the *x*‐axis and *gradus suavitatis* (C). Correlation between the *y*‐axis and interval size (D).

The *x* coordinates did not correlate with audio‐based roughness’ metric in the complete sample (*ρ* = −0.34, *p *= 0.287) nor in the English/Mandarin‐speakers’ groups separately (*ρW* = −0.42, *p* = 0.168; *ρE* = −0.23, *p* = 0.457). However, they were highly correlated with the symbolic metric of roughness (*ρ* = −0.77, *p* = 0.005, see Figure 5) and with *gradus suavitatis* (*ρ* = −0.77, *p* = 0.003, see Figure 5) in the overall sample (*gradus suavitatis* resulted in being highly correlated with the symbolic metric of roughness, *ρ* = 0.83, *p* < 0.001). As for the *y* coordinates of the MDS, they seem to imply a measure of the intervals’ size. Thus, we correlated them with a measure of the interval size in semitones assuming octave equivalence (Distance_mod_ = Semitone count mod 12), finding a very sound correlation (*ρ* = 0.85, *p* < 0.001, see Figure 5). This correlation was stronger in the Western sample as opposed to the Eastern (*ρ_W_* = 0.93, *p* < 0.001; *ρ_E_* = 0.75, *p* = 0.006).

### 3.4 Principal Component Analysis

**FIGURE 6:**
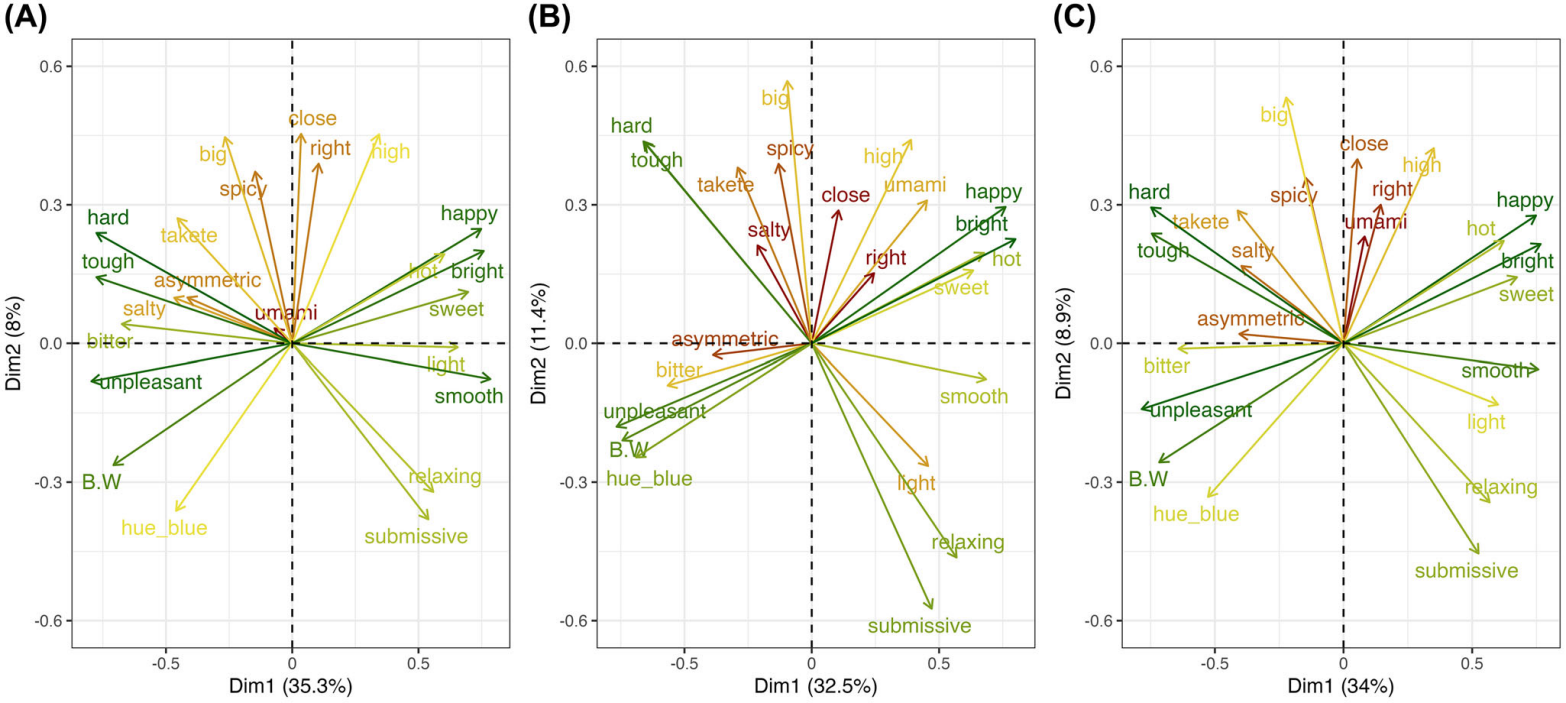
The English‐speakers (A) and Mandarin‐speakers (B) samples show very similar principal component analysis (PCA) solutions, with comparable orientations of ratings across the two dimensions, thus indicating a consistent perceptual structure across groups. The similarity across samples is reflected in the overall PCA solution (C). It is worth stressing that the PCA configuration displays a star‐like structure, suggesting that the overall pattern is not driven by a single dominant dimension. Colors correspond to the cos^2^ values, which express the quality of representation of each variable on the PCA axes. The gradient ranges from red (low values) to green (high values), with yellow indicating intermediate values.

Figure 6 shows the PCA of the sensory and affective dimensions for the English‐speaking (A), Mandarin‐speaking (B), and overall (C) samples. Across samples, the first two components accounted for a comparable proportion of variance (Dim1 ≈ 32%–35%; Dim2 ≈ 9%–11%) and revealed a highly similar dimensional organization. A closer visual inspection suggests that affective and hedonic dimensions (e.g., happy, relaxing) contribute prominently to the first component (*x*‐axis) but do not fully determine the spatial structure. Several perceptual and sensory descriptors (e.g., height, size) project along partially independent directions, suggesting additional dimensions beyond a purely hedonic axis. In particular, the second component (*y*‐axis) seems to capture variations in perceived intensity or sensory salience, partially orthogonal to hedonic evaluation. The cosine‐squared values indicate that not all ratings are equally well represented by the first two components, consistent with a multidimensional organization of responses rather than a single evaluative dimension. Notably, this overall pattern is qualitatively consistent across English‐ and Mandarin‐speakers.

## 4 Discussion

This study reports an investigation of the multisensory mappings between musical intervals and various sensory and affective qualities in two samples, one comprising participants from the United Kingdom and North America who completed the study in English, and another comprising participants from China who completed the study in Mandarin. Notably, despite differences between the two samples in terms of musical training, the findings reveal consistent cross‐group mappings between musical intervals and a range of gustatory, visual, tactile, and emotional attributes. Furthermore, MDS analysis demonstrated that these associations led to a clear partitioning of the harmonic space into two distinct regions, that is, consonance and dissonance. These categories emerged as the primary underlying factors mediating the observed mappings in both groups.

The strength and prevalence of significant relationships between intervals and multisensory/emotional attributes varied across domains. Emotional attributes showed the largest proportion of significant associations, followed by tactile and visual (color/shape) domains, with gustatory mappings being somewhat less pronounced. In contrast, only a few spatial and physical features exhibited significant relationships with musical intervals. This pattern likely reflects the well‐established salience of emotional responses in music perception [56]. It also mirrors the predominance of crossmodal associations involving spatially grounded senses such as vision and touch, compared to the less consistent or weaker links with chemical senses, particularly taste [1, 57].

When the participants were presented with contrasting dimensions (e.g., low/high) or unipolar dimensions (e.g., bitter), most ratings were polarized on the basis of the consonant or dissonant nature of the musical stimuli (see Costa et al. [29], for similar findings using emotional descriptors). Specifically, consonant intervals tend to be perceived as lighter, happier, more pleasant, softer, more tender, more relaxing, smoother, more *maluma*, more colorful, hotter, and more symmetric. In contrast, dissonant intervals were perceived as bigger, heavier, sadder, more unpleasant, tougher, more dominant and arousing, harder, rougher, colder, darker, more *takete*, more black‐and‐white, and more asymmetric.

Nearly all variance in the data can be accounted for by consonance and dissonance alone. The two dimensions of the MDS analysis likely represent distinct perceptual qualities of musical intervals. Specifically, in Figure 4, the horizontal axis appears to reflect the degree of consonance, with more consonant intervals positioned on the right and more dissonant ones on the left, as suggested by the strong correlation between the horizontal axis and consonance indices such as roughness and Euler’s *gradus suavitatis* [58, 59]. Meanwhile, the vertical axis corresponds to interval size, with intervals tending to increase in size from bottom to top, except for the octave.

The prominence of consonance–dissonance as the principal axis of multisensory and emotional mappings suggests that this dimension may serve as a core organizing principle in the cognitive representation of musical structure. This interpretation aligns with a substantial body of research indicating that the perception of consonance and dissonance is grounded in neurobiological mechanisms [60, 61, 62, 63, 64, 65]. These results demonstrate that a single, musically intrinsic feature (i.e., harmonic stability) can predict cross‐sensory mappings across multiple modalities beyond the auditory domain.

Consonance and dissonance are commonly defined in terms of the perceptual effects they generate in listeners, with consonances typically perceived as smooth, stable, and harmonious, and dissonances as rough, tense, or unstable [62]. Considerable debate remains regarding the primary mechanisms underlying consonance perception, as well as the respective roles of biological predispositions and culturally situated musical practices. Recent work has increasingly suggested that consonance and dissonance emerge from interactions between perceptual constraints and cultural exposure [66, 67, 68]. Studies by Lahdelma and Eerola [69, 70] showed that judgments of consonance and dissonance do not merely reflect or mirror the commonly assumed related constructs of pleasantness or preference, indicating that they likely index partially distinct perceptual dimensions or factors. In this line, the comprehensive review by Harrison and Pearce [53] proposed that consonance judgments are best explained as a combination of chord harmonicity, roughness, and familiarity. Subsequent studies have largely supported and further refined these conclusions [71, 72, 73]. That being said, for the purposes of the present paper, consonance and dissonance are primarily used as descriptive categories for identifying groups of musical intervals that emerged from MDS, with no implications regarding the ultimate origins or mechanisms underlying consonance perception.

When explaining the data based on the hypotheses that are currently discussed in the literature to date [1, 74], the affective mediation hypothesis appears to be the strongest candidate to account for many of the consistent mappings that emerged in our study [75]. According to this account, crossmodal associations might be explained by the similar emotional profile, or meaning, of the stimuli that happen to be associated [76, 77]. For instance, colors and musical excerpts might be consensually matched by observers due to the shared emotional meaning [67]. This hypothesis would be supported by the emotional attributes ascribed to or perceived in musical intervals [27, 29, 61, 78] and the clear emotional connotations of many investigated features, such as rough/smooth, *maluma/takete*, and sweet/salty [26, 79, 80]. For instance, our finding indicating that consonances (i.e., major thirds, perfect fourths and fifths, major and minor sixths, and octaves) were more frequently associated with yellow hues may align with the studies demonstrating that such warm hues are typically rated as pleasant [10, 81, 82]. However, the color‐emotion literature also indicates that short‐wavelength hues (e.g., blue–purple) often receive positive valence ratings, which complicates a straightforward interpretation. Adding further nuance, evidence suggests that warm–cool judgements correlate with like–dislike ratings in British observers but not in Chinese observers [83]. Furthermore, the literature on the aesthetic/hedonic appreciation of other sensory qualities, such as “salty” and “B&W,” reports mixed and often context‐dependent findings [84, 85, 86, 87], thereby precluding a unidirectional pleasant/unpleasant interpretation of our results.

A PCA was conducted to further refine the interpretation of the results, particularly with respect to the role of pleasantness and unpleasantness. The first PCA dimension accounted for approximately one‐third of the total variance, suggesting the presence of a meaningful hedonic or affective component in the data. This is perhaps unsurprising, given that many of the sensory descriptors used in the study can themselves be broadly organized along a positive–negative evaluative dimension.

Importantly, however, the starred configuration observed in the PCA plots indicates that the structure of the data cannot be straightforwardly reduced to a single pleasant–unpleasant axis. Several descriptors—including close, big, spicy, high, submissive, relaxing, and hard—appear nearly orthogonal to the hedonic dimension in at least one of the PCA solutions (English‐speaking, Mandarin‐speaking, or overall). Moreover, the central finding of the present study is that consonance and dissonance emerge as major organizing principles of the observed multisensory associations, and these dimensions do not simply reduce to or align with pleasantness and unpleasantness. We, therefore, adopt a more cautious interpretation, suggesting that the observed structure likely reflects the interaction of multiple factors, including hedonic, auditory, and acoustic properties.

Another possible explanation draws on the literature highlighting the role of conceptual metaphors in structuring listeners’ multisensory associations. Within this framework, crossmodal correspondences are grounded in cross‐domain metaphors that reflect broader conceptual structures, rather than solely through affective mediation [88]. Previous research has demonstrated systematic crossmodal mappings between musical parameters and non‐auditory sensory domains. In particular, Eitan et al. have shown that listeners consistently associate musical pitch with spatial, visual, and tactile dimensions, such as height, brightness, and sharpness, suggesting that musical perception is structured by networks of cross‐domain correspondences [77, 89]. These mappings have been interpreted as reflecting underlying conceptual metaphors linking auditory features to spatial and embodied schemas [90]. The present study extends this line of research by investigating whether such multisensory mappings are systematically organized by the consonance–dissonance dimension across a broader range of sensory and affective attributes. However, because language plays a central role in shaping how sensory experiences are conceptualized [91], this account must also consider cross‐linguistic differences in how such metaphors are expressed (e.g., in English and Mandarin), and whether comparable conceptual mappings underpin the associations in each language.

Our study might contribute also to the ongoing debate on the origins of foundational constructs in harmony—that is, consonance and dissonance—where conflicting perspectives have often emerged [66, 92, 93]. The present results align with findings on shared traits in both music perception and crossmodal correspondences involving auditory stimuli [69, 94, 95, 96]. However, although the difference in terms of musical training did not affect the multisensory mappings, the data collected on musical background do not allow us to clearly determine the extent to which the two groups differ in their exposure to Western music and harmony.

One limitation of the present study is the use of linguistic labels for tastes and odors rather than actual tastants or odors. Consequently, participants’ associations may have been influenced by the linguistic properties of the food names themselves (e.g., the roundedness or angularity of the graphemes [97]). Future research should therefore incorporate more ecologically valid stimuli to directly assess sensory mappings and eliminate potential confounds related to the presentation of verbalized sensory features [25, 98]. Another potential limitation concerns the absence of detailed data on bilingualism and English proficiency among the Chinese participants, and specific non‐linguistic cultural practices, which could have provided additional informative variables for examining cross‐group differences more closely. Furthermore, all musical stimuli were presented using a piano timbre. Given the role of timbre in shaping crossmodal associations with complex musical stimuli [88], future research could explore how different timbres influence multisensory mappings of simple musical stimuli, such as harmonic intervals. Finally, given the relatively narrow pitch range used in the present study, the interaction between interval structure, pitch register, and crossmodal mappings was not directly addressed here and therefore remains an important question for future research.

The present findings may offer several potential avenues for application. First, the multisensory mappings could be leveraged in crossmodally augmented artistic practices, where sound is systematically paired with visual, tactile, or gustatory elements to enhance aesthetic experience [99]. Relatedly, the mappings between intervals and dimensions of the chemical senses could provide a reliable basis for the sonification of olfactory and gustatory experiences. For instance, mapping fragrances, food textures, or wine profiles onto harmonic structures in ways that are perceptually meaningful and systematically grounded [100, 101]. Second, they offer a principled basis for the design of sensory substitution systems, in which auditory signals could convey information typically accessed through other modalities (e.g., translating visual features into structured harmonic patterns) [102]. Third, these correspondences may inform sensory augmentation technologies, enabling more robust mapping across senses in a systematic manner [103].

## 5 Conclusion

This study shows that the crossmodal mapping of musical intervals onto sensory and affective dimensions is predominantly structured by a musical factor, namely consonance and dissonance. Unlike previous research, which has largely focused on isolated sensory correspondences (e.g., pitch‐brightness or pitch‐taste), these findings demonstrate a robust, domain‐general principle in which consonance and dissonance structure multiple sensory mappings. The fact that no explicit mention of consonance/dissonance was made in the experiment, yet participants’ responses consistently aligned with this dimension, suggests that these associations might well be perceptually grounded and shared across the two groups of participants, namely, English‐speakers from North America and the United Kingdom and Mandarin‐speakers from China. This provides a new perspective on the multisensory dimension of music perception, supporting the idea that musical consonance and dissonance reflect deeper cognitive and perceptual regularities that extend across sensory systems.

## Author Contributions

Nicola Di Stefano: conceptualization, methodology, writing – original draft, funding acquisition. Alessandro Ansani: methodology, writing – original draft, formal analysis, funding acquisition. Andrea Schiavio: methodology, writing – original draft. Suvi Saarikallio: methodology, writing – original draft, funding acquisition. Petri Toiviainen: methodology, formal analysis, writing – original draft, funding acquisition. Elvira Brattico: methodology, writing – original draft. Charles Spence: conceptualization, methodology, supervision. All authors read and approved the final version of the manuscript.

## Conflicts of Interest

The authors declare no conflicts of interest.

## Supporting information




**Supporting Information**: nyas70337‐sup‐0001‐SuppMat.docx

## Data Availability

The data and musical stimuli that support the findings of this study are openly available in OSF at https://osf.io/8nxr7.
