# Supplementary material for: Consonance Shapes the Multisensory and Emotional Mappings of Musical Intervals Across English‐ and Mandarin‐Speakers
Source: Ann N Y Acad Sci. 2026 Jul 23;1561(1):e70337. doi: 10.1111/nyas.70337 (PMC13395244; doi:10.1111/nyas.70337)
Supplement: Supplementary file 1 — Supporting Information: nyas70337‐sup‐0001‐SuppMat.docx [file NYAS-1561-0-s001.docx]

**Supplementary Information for**

**Consonance shapes the multisensory and emotional mappings of musical intervals across English- and Mandarin-speakers**

Nicola Di Stefano, Alessandro Ansani, Andrea Schiavio, Suvi Saarikallio,

Petri Toiviainen, Elvira Brattico, & Charles Spence

**Nicola Di Stefano:** [**nicola.distefano@istc.cnr.it**](mailto:nicola.distefano@istc.cnr.it)

**This file includes:**

**Table S1**

**Figures S1 to S8**

**Table S1**. Fit indices of the Generalised Linear Mixed Models comparisons

| **Name** | **Model** | **AIC** | **BIC** | **ΔAIC** | **ΔBIC** | ***BF*** | **Model** |
| --- | --- | --- | --- | --- | --- | --- | --- |
| Spicy | Simple | 20703 | 20790 | -15 | -86 | 3.60 × 10^18^ | GLMM |
|  | Complex | 20718 | 20876 |  |  |  |  |
| Sweet | Simple | 21021 | 21109 | 2 | -67 | 4.40 × 10^14^ | GLMM |
|  | Complex | 21019 | 21176 |  |  |  |  |
| Salty | Simple | 21359 | 21446 | 29 | -41 | 7.69 × 10^8^ | GLMM |
|  | Complex | 21330 | 21487 |  |  |  |  |
| Bitter | Simple | 21566 | 21653 | -9 | -79 | 1.44 × 10^17^ | GLMM |
|  | Complex | 21575 | 21732 |  |  |  |  |
| Umami | Simple | 21347 | 21434 | 52 | -18 | 6957 | GLMM |
|  | Complex | 21295 | 21452 |  |  |  |  |
| Far/close | Simple | 22322 | 22409 | 5 | -66 | 1.79 × 10^14^ | LMM |
|  | Complex | 22317 | 22475 |  |  |  |  |
| Left/right | Simple | 21432 | 21520 | -13 | -82 | 7.82 × 10^17^ | LMM |
|  | Complex | 21445 | 21602 |  |  |  |  |
| Low/high | Simple | 22039 | 22126 | -7 | -77 | 5.97 × 10^16^ | LMM |
|  | Complex | 22046 | 22203 |  |  |  |  |
| Small/big | Simple | 21649 | 21736 | 1 | -69 | 9.10 × 10^14^ | LMM |
|  | Complex | 21648 | 21805 |  |  |  |  |
| Heavy/light | Simple | 21942 | 22029 | 21 | -49 | 3.73 × 10^10^ | LMM |
|  | Complex | 21921 | 22078 |  |  |  |  |
| Symmetric/  asymmetrical | Simple | 22193 | 22280 | -7 | -77 | 4.50 × 10^16^ | LMM |
|  | Complex | 22200 | 22357 |  |  |  |  |
| Sad/happy | Simple | 21606 | 21693 | 6 | -65 | 9.60 × 10^13^ | LMM |
|  | Complex | 21600 | 21758 |  |  |  |  |
| Pleasant/  unpleasant | Simple | 21940 | 22027 | 37 | -34 | 1.88 × 10^7^ | LMM |
|  | Complex | 21903 | 22061 |  |  |  |  |
| Tender/tough | Simple | 21672 | 21759 | 12 | -59 | 4.50 × 10^12^ | LMM |
|  | Complex | 21660 | 21818 |  |  |  |  |
| Dominant/  submissive | Simple | 21916 | 22004 | -10 | -80 | 4.50 × 10^17^ | LMM |
|  | Complex | 21926 | 22084 |  |  |  |  |
| Arousing/  relaxing | Simple | 21435 | 21522 | -6 | -77 | 3.83 × 10^16^ | LMM |
|  | Complex | 21441 | 21599 |  |  |  |  |
| Soft/hard | Simple | 21974 | 22061 | 15 | -55 | 1.03 × 10^12^ | LMM |
|  | Complex | 21959 | 22116 |  |  |  |  |
| Rough/smooth | Simple | 21868 | 21955 | 17 | -53 | 2.87 × 10^11^ | LMM |
|  | Complex | 21851 | 22008 |  |  |  |  |
| Cold/hot | Simple | 21484 | 21572 | -9 | -79 | 1.28 × 10^17^ | LMM |
|  | Complex | 21493 | 21651 |  |  |  |  |
| Dark/bright | Simple | 21859 | 21947 | -5 | -74 | 1.19 × 10^16^ | LMM |
|  | Complex | 21864 | 22021 |  |  |  |  |
| Maluma/takete | Simple | 22876 | 22963 | 10 | -60 | 1.03 × 10^13^ | LMM |
|  | Complex | 22866 | 23023 |  |  |  |  |
| Color/B&W | Simple | 22622 | 22709 | -9 | -79 | 1.89 × 10^17^ | LMM |
|  | Complex | 22631 | 22788 |  |  |  |  |
| Hue (>cold) | Simple | 40505 | 40592 | 7 | -63 | 5.61 × 10^13^ | LMM |
|  | Complex | 40498 | 40655 |  |  |  |  |


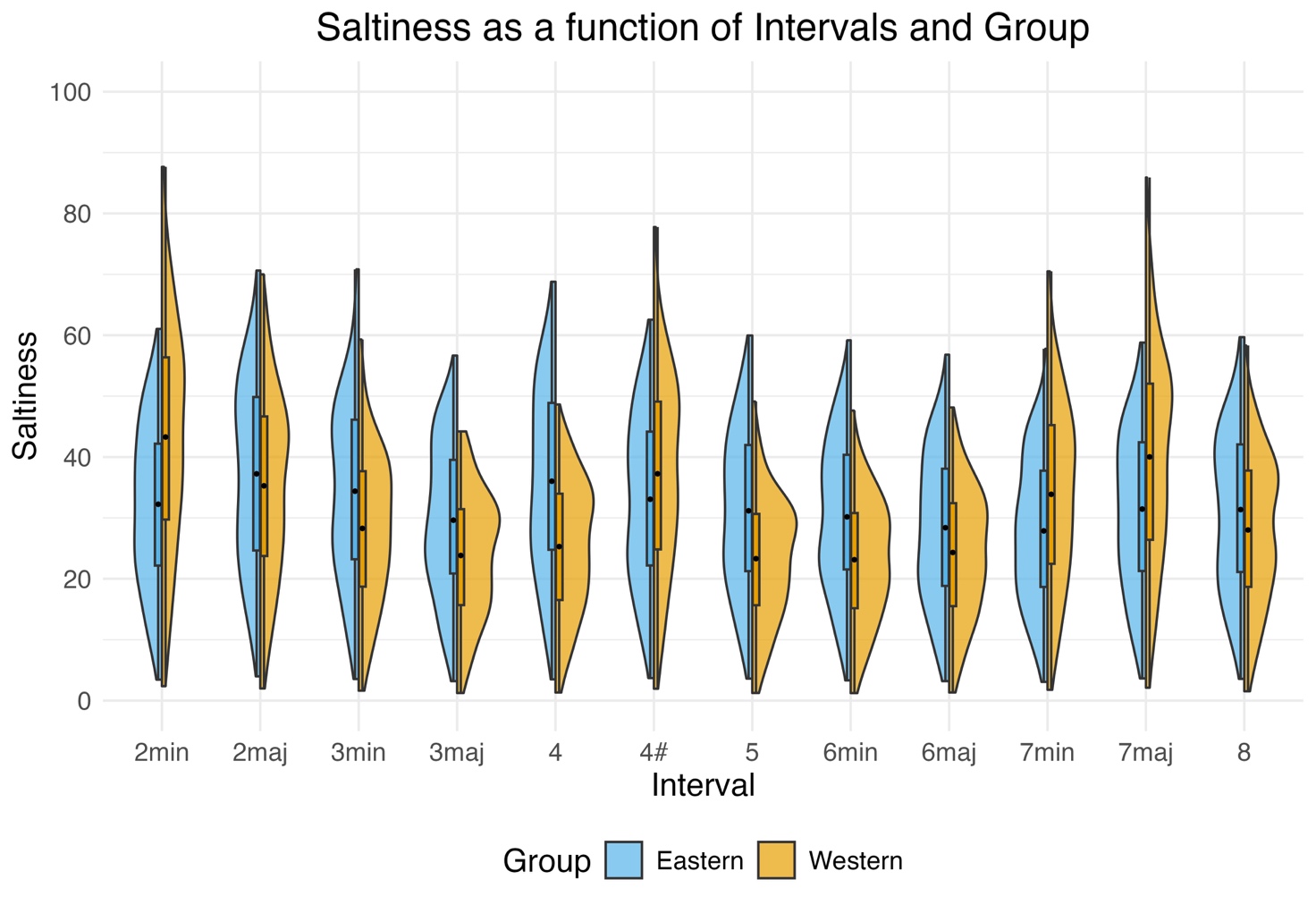


**Fig. S1.** Saltiness as a function of interval and group. In the y-axis, 100 indicates that participants rated the interval as salty, while 0 indicates that the interval is perceived as not-salty. The form of the violin plots indicates the distribution curve. The boxplots within each violin represent interquartile ranges (IQRs). Black dots within the boxplots indicate mean values. Colours represent groups, namely, Mandarin-speakers (pale blue) and English-speakers (orange).

**
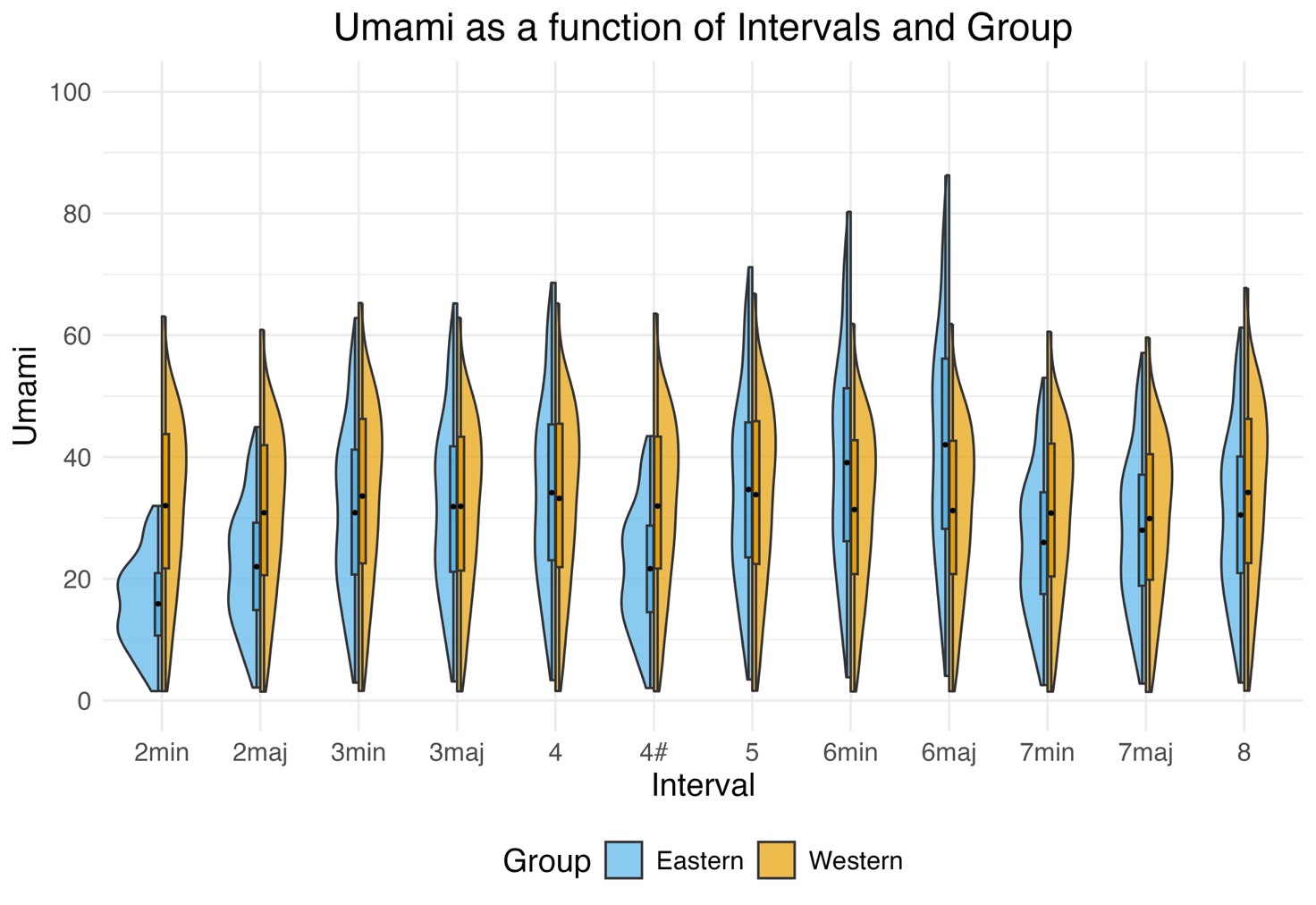
**

**Fig. S2.** Umami as a function of interval and group. In the y-axis, 100 indicates that participants rated the interval as umami, while 0 indicates that the interval is perceived as not-umami. The form of the violin plots indicates the distribution curve. The boxplots within each violin represent interquartile ranges (IQRs). Black dots within the boxplots indicate mean values. Colours represent groups, namely, Mandarin-speakers (pale blue) and English-speakers (orange).


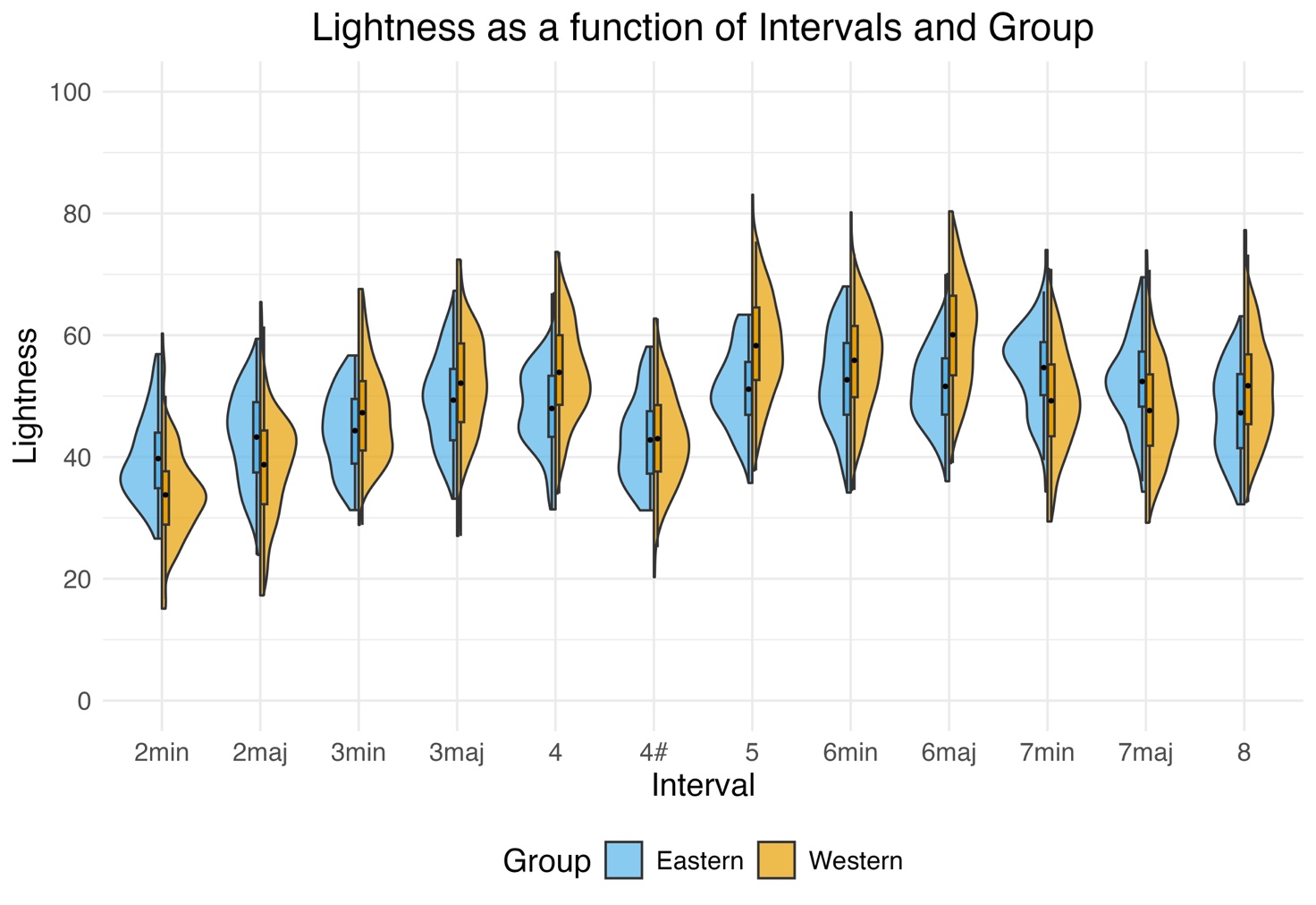


**Fig. S3.** Lightness as a function of interval and group. In the y-axis, 100 indicates that participants rated the interval as light, while 0 indicates that the interval is perceived as dark. The form of the violin plots indicates the distribution curve. The boxplots within each violin represent interquartile ranges (IQRs). Black dots within the boxplots indicate mean values. Colours represent groups, namely, Mandarin-speakers (pale blue) and English-speakers (orange).


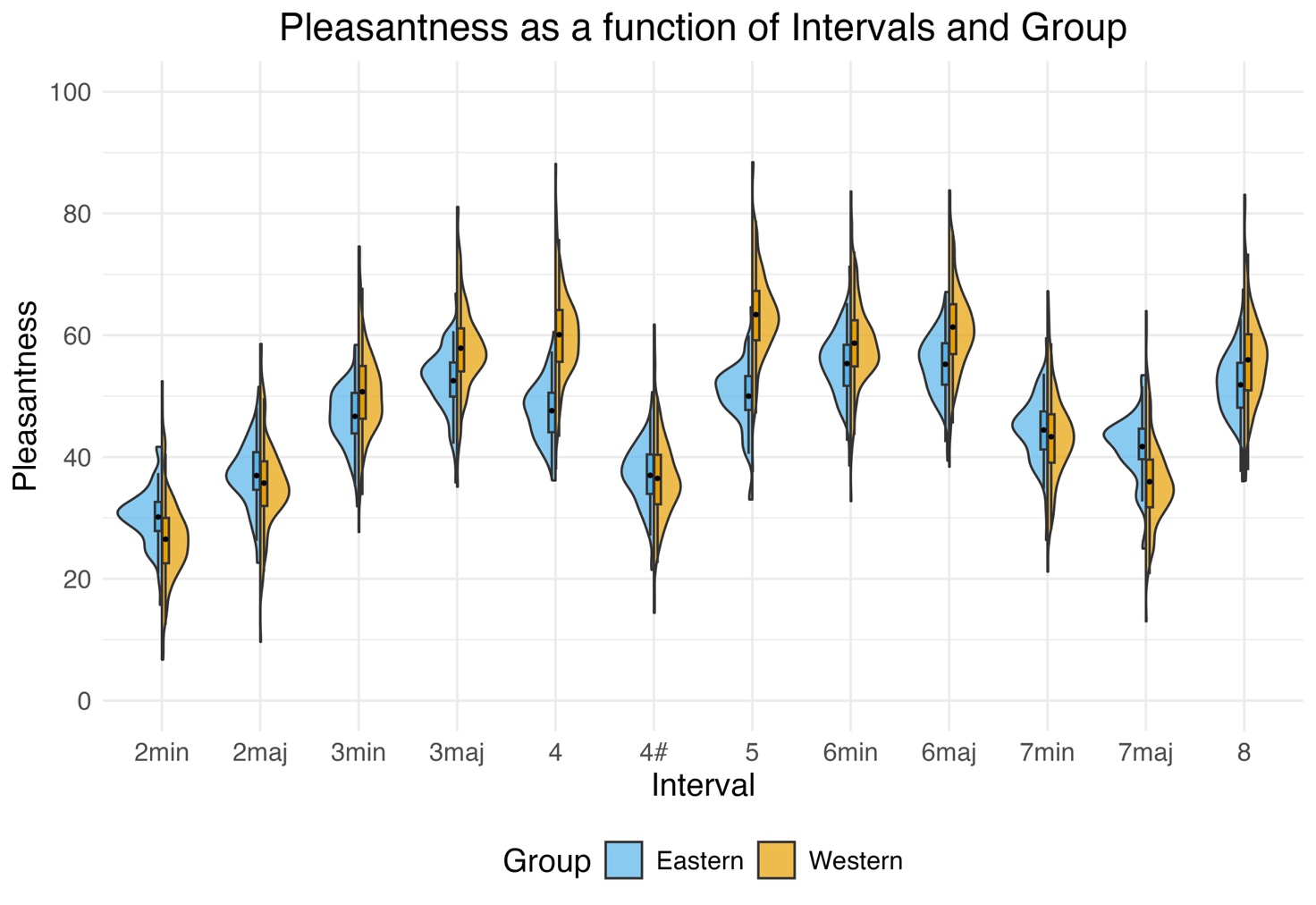


**Fig S4.** Pleasantness as a function of interval and group. In the y-axis, 100 indicates that participants rated the interval as pleasant, while 0 indicates that the interval is perceived as unpleasant. The form of the violin plots indicates the distribution curve. The boxplots within each violin represent interquartile ranges (IQRs). Black dots within the boxplots indicate mean values. Colours represent groups, namely, Mandarin-speakers (pale blue) and English-speakers (orange).


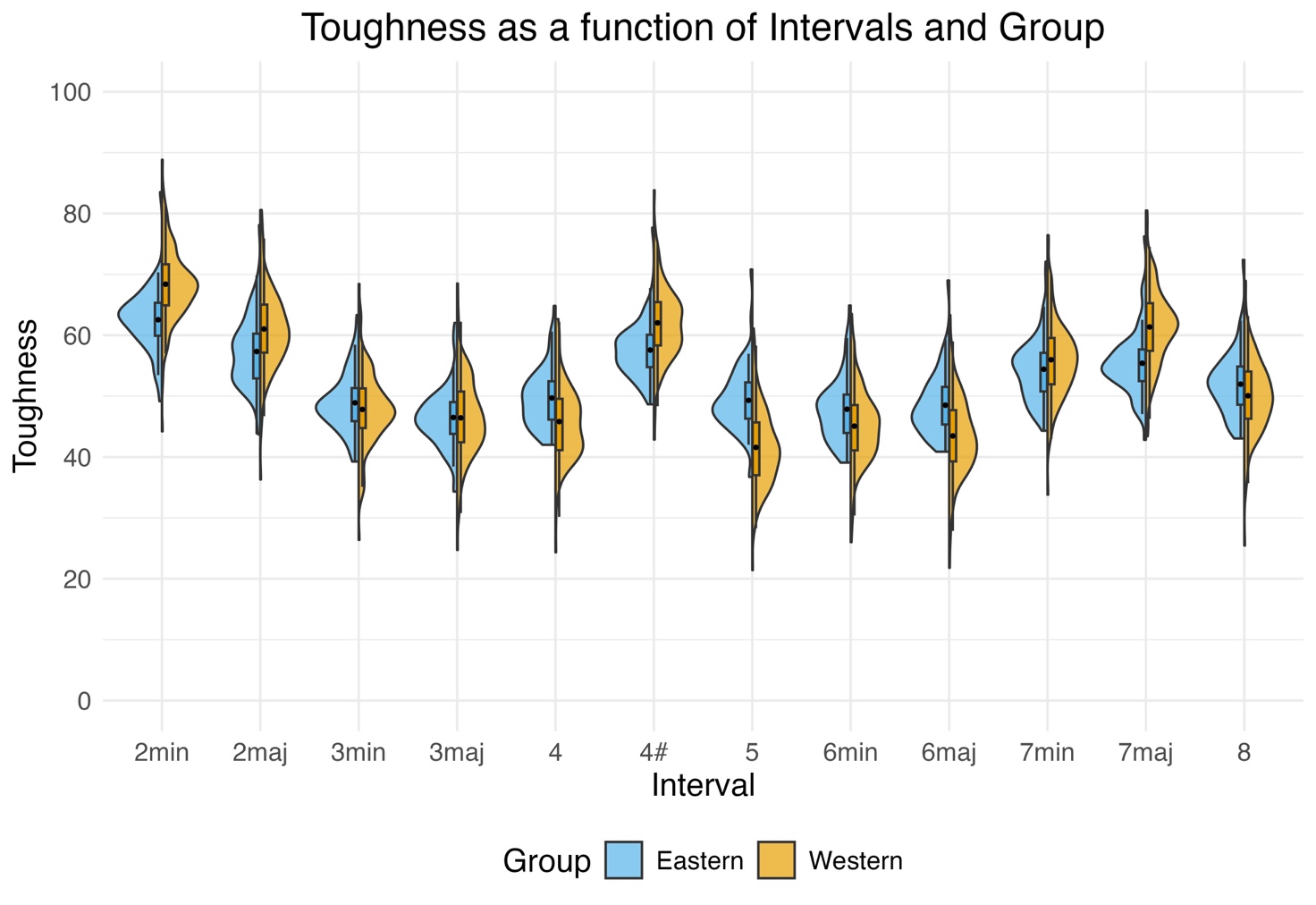


**Fig S5**. Toughness as a function of interval and group. In the y-axis, 100 indicates that participants rated the interval as tough, while 0 indicates that the interval is perceived as tender. The form of the violin plots indicates the distribution curve. The boxplots within each violin represent interquartile ranges (IQRs). Black dots within the boxplots indicate mean values. Colours represent groups, namely, Mandarin-speakers (pale blue) and English-speakers (orange).


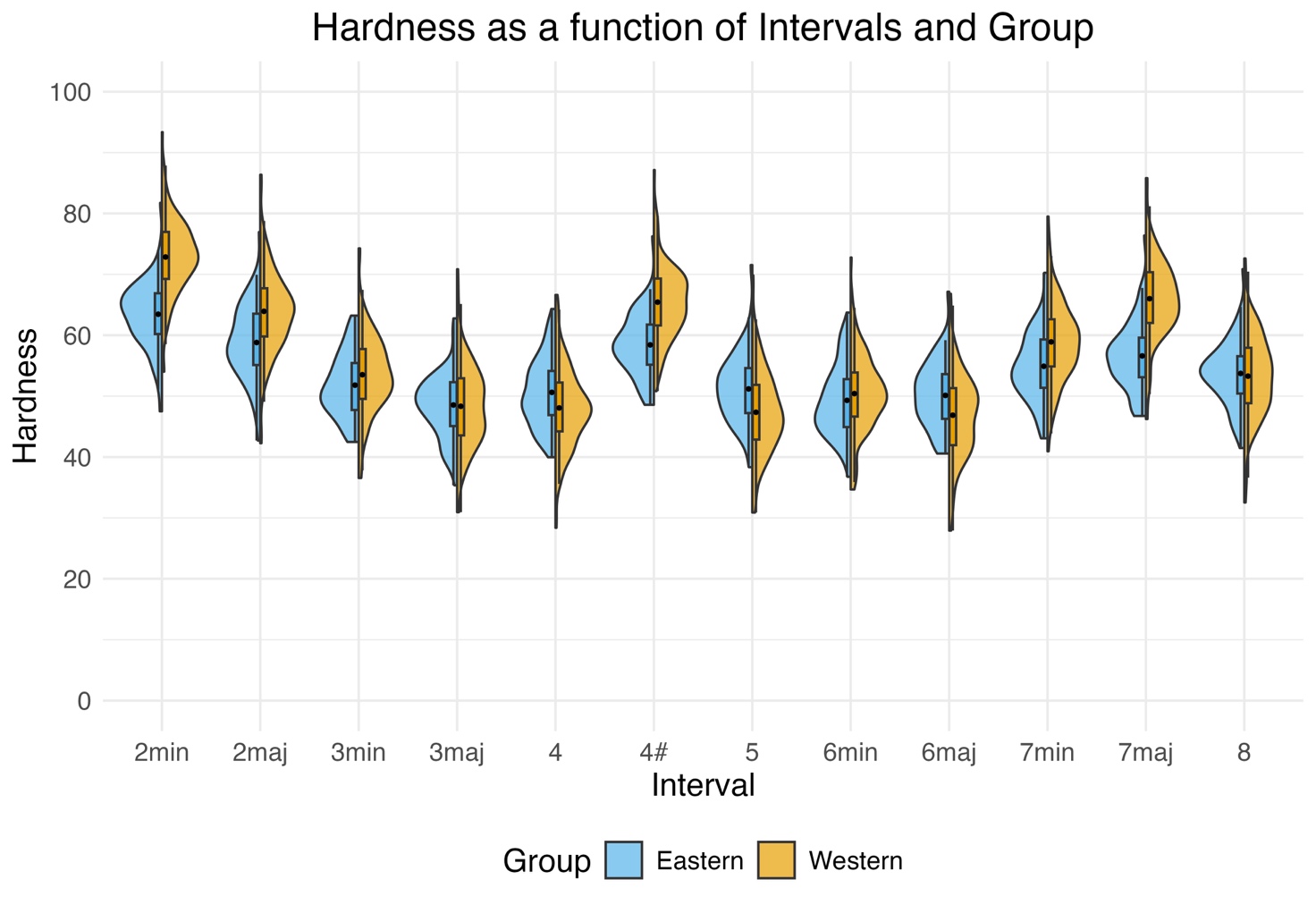


**Fig S6**. Hardness as a function of interval and group. In the y-axis, 100 indicates that participants rated the interval as hard, while 0 indicates that the interval is perceived as soft. The form of the violin plots indicates the distribution curve. The boxplots within each violin represent interquartile ranges (IQRs). Black dots within the boxplots indicate mean values. Colours represent groups, namely, Mandarin-speakers (pale blue) and English-speakers (orange).


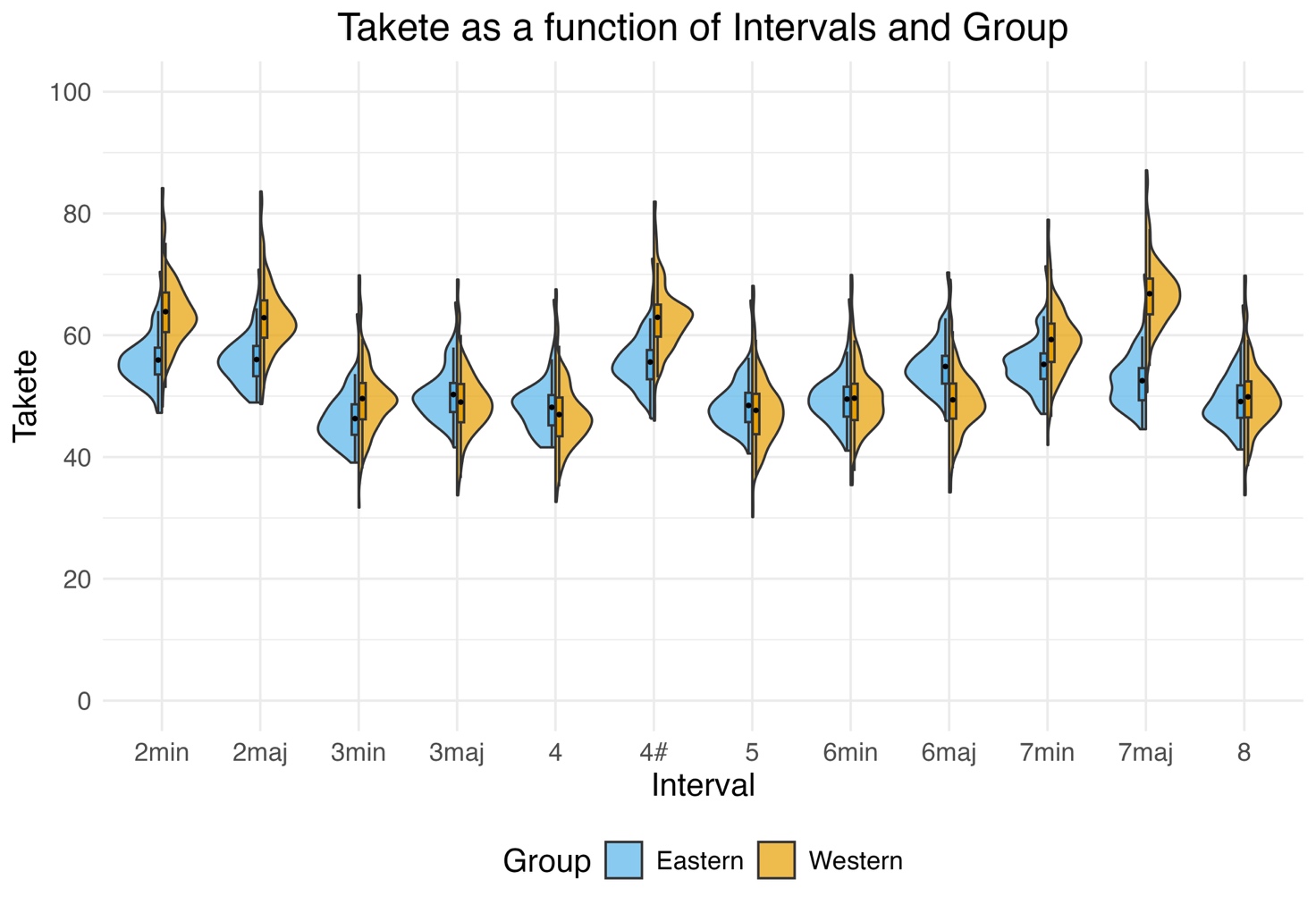


**Fig S7.** Angularity (or ‘Takete’) as a function of interval and group. In the y-axis, 100 indicates that participants rated the interval as angular (takete), while 0 indicates that the interval is perceived as round (maluma). The form of the violin plots indicates the distribution curve. The boxplots within each violin represent interquartile ranges (IQRs). Black dots within the boxplots indicate mean values. Colours represent groups, namely, Mandarin-speakers (pale blue) and English-speakers (orange).


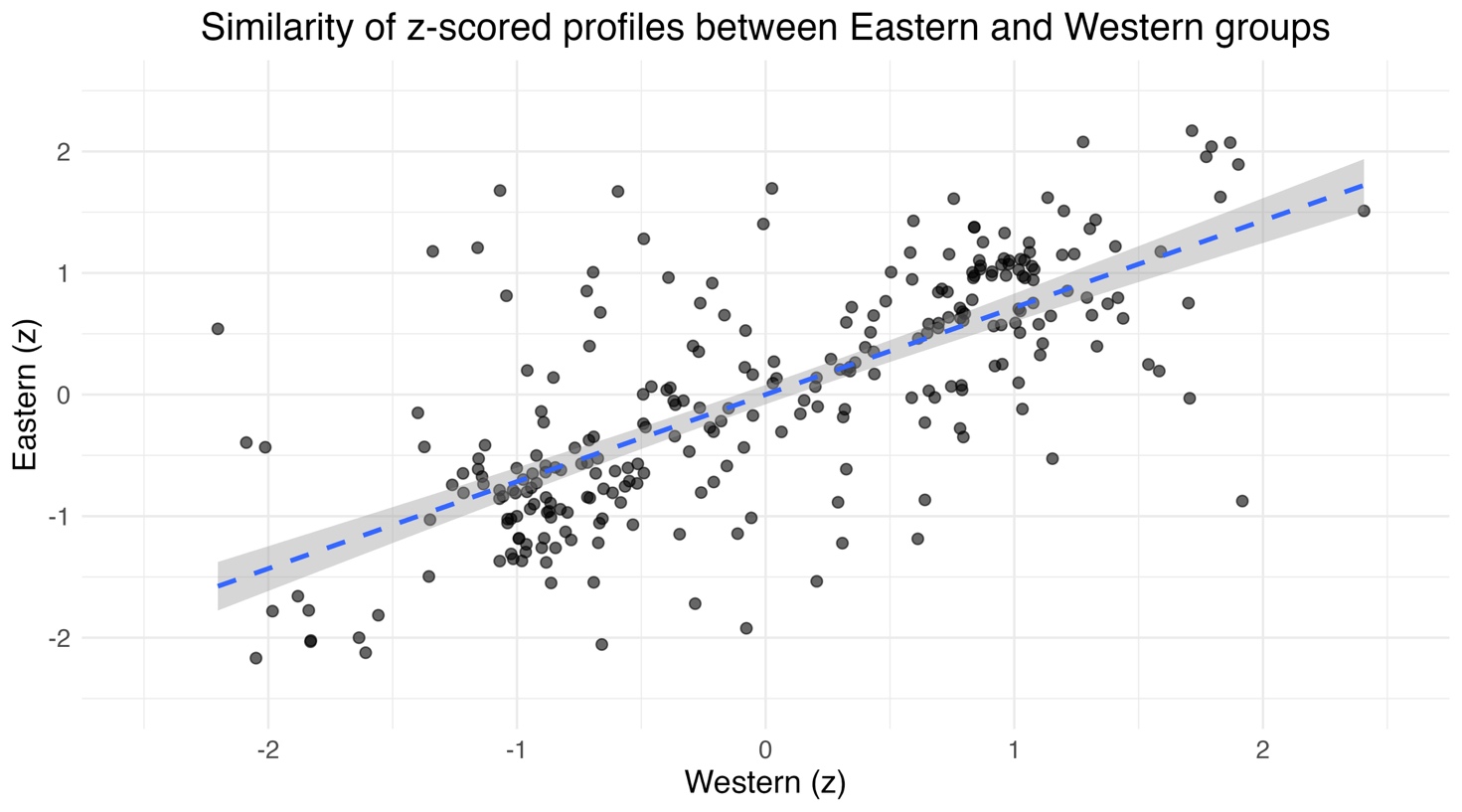


**Fig S8.** Plot of the Z-scored profiles of the Western (x axis) and Eastern (y axis) listeners. Each point corresponds to an interval × dimension cell derived from the vectorised matrices of mean ratings. The dashed blue line represents the best-fitting linear association, with the shaded band indicating the 95% confidence interval.
